# The ideal harvest time for seeds of hybrid maize (*Zea mays* L.) XY335 and ZD958 produced in multiple environments

**DOI:** 10.1038/s41598-017-16071-4

**Published:** 2017-12-13

**Authors:** Riliang Gu, Li Li, Xiaolin Liang, Yanbo Wang, Tinglu Fan, Ying Wang, Jianhua Wang

**Affiliations:** 1 Center of Seed Science and Technology, Beijing Key Laboratory of Crop Genetics and Breeding, Beijing Innovation Center for Seed Technology (MOA), College of Agronomy and Biotechnology, China Agricultural University, Beijing, 100193 China; 2 Institute of Grain Crops, Xinjiang Academy of Agricultural Sciences, Urumqi, 830091 China; 3 Maize Research Institute, Liaoning Academy of Agricultural Sciences, Shenyang, 110161 China; 4 Gansu Academy of Agricultural Sciences, Lanzhou, 730070 China; 5 Gansu Jiuquan Agricultural Research Academy, Jiuquan, 735000 China

## Abstract

To identify the ideal harvest time (IHT) for the seed production of XY335 and ZD958, six seed-related traits were evaluated in seeds harvested at 11 harvest stages in 8 environments. Standard germination (SG), accelerated aging germination (AAG) and cold test germination (CTG) were vigor traits; hundred-seed weight (HSW) and seed moisture content (SMC) were physiological traits; and ≥10 °C accumulated temperature from pollination to harvest (AT10_ph_) was an ecological trait. All the traits were significantly affected by harvest stage. The responses of SG, AAG, CTG and HSW to postponing harvest stage fit quadratic models, while SMC and AT10_ph_ fit linear models. The IHT (indicated by the last date to reach maximum SG, AAG and CTG) were 57.97 DAP and 56.80 DAP for XY335 and ZD958, respectively. SMC and AT10_ph_ at IHT were 33.15% and 1234 °C for XY335, and 34.98% and 1226 °C for ZD958, respectively. The period to reach the maximum HSW was 5 days later than the IHT. Compared to HSW and SMC, AT10_ph_ had a closer relationship to the seed vigor traits. Together with the fact that AT10_ph_ was less affected by environment, these results suggested that AT10_ph_ may be a novel indicator for determining the IHT.

## Introduction

Along with rice and wheat, maize is one of the three major cereal crops in the world. In China, hybrid maize composes more than 95% of the maize cultivation^1^, and the annual demand for hybrid seeds is approximately 1.1 billion kg^2,3^. Producing high-quality hybrid maize seeds is extremely important for the economic benefit of seed companies, as well as the demand for Chinese food security.

Seed quality is generally reflected by seed vigor, a comprehensive term that refers to the total properties of seed activities from germination under broad environments to seed longevity during storage. Standard germination (SG), cold test germination (CTG) and accelerated aging test germination (AAG) are the three most widely used parameters regarding seed vigor^4^. SG is seed germination conducted under favorable conditions; it usually overestimates the actual germination in most fields in suboptimal conditions. CTG and AAG are two seed vigor indexes that have been used for predicting field seedling emergence^5,6^. A previous study indicated that the predictive value of SG is relatively low (8.3%), while the value of AAG is moderate (46.7%) in a less optimal condition^7^.

Harvesting at the proper time is a key factor that contributes to obtaining high vigor seeds in production. Harvesting too early may result in immature seeds that have poor vigor^8^. On the other hand, the delayed harvest of seeds increases the potential damages from insects and microorganisms that may accelerate the seed deterioration process^8,9^. A delay in harvest also increases the potential damage from fall frost or excessive rain during seed post-harvest processes^10^. Therefore, studies to determine the ideal harvest time (IHT) for seed production are necessary to ensure maximum vigor in hybrid maize.

Morphological and physiological changes that occur during the maturation process have been used as parameters to identify IHT in maize. The parameters frequently used are black layer formation, milkline development, seed weight (usually expressed as hundred-seed weight, HSW) and seed moisture content (SMC)^11–15^. Black layer formation and milkline development are two characteristics easily detected in the field and usually serve as maturity indicators in practices. The SMC decreases along with the seed maturity process and has also been widely used to determine seed maturity. However, some researchers concluded that the above three indicators were not always reliable since they varied among genotypes, environments and sowing dates^12,13,16–18^. Meanwhile, HSW was suggested as a reliable indicator since seeds always reached maximum vigor when they achieved maximum weight (also called physiological maturity, PM)^19,20^. However, previous studies mostly focused on the relationship between HSW and seed vigor of SG, but little research has been reported on seed vigor under CTG and AAG^20^.

Thermal conditions are important for regulating crop growth. In 1730, Reaumut first introduced the concept of heat units, or thermal time, into agricultural sciences, and many methods have been developed for calculating thermal time^21^. In China, the ≥10 °C accumulated temperature (AT10) was routinely used as an indicator of thermal conditions during growth periods in crop ecology^22–25^. In terms of seed production, although thermal condition is an important ecological factor that influences seed maturity, the utilization of AT10 from pollination to harvest (AT10_ph_) as an indicator for seed maturity has not been published since the relationship between AT10_ph_ and seed maturity is unclear.

Gansu province and the Xinjiang Uygur autonomous region are the two largest seed producers, providing approximately 39% and 23% of the hybrid maize seeds produced in China, respectively^3^. Liaoning province is another important seed-producing area located in Northeast China^26^. XY335 and ZD958 are currently the most widely cultivated hybrids in China. In this study, we investigated the seed vigor indexes of SG, CTG and AAG and the seed maturity parameters of HSW, SMC and AT10_ph_ in both XY335 and ZD958 hybrids in different harvest stages at the above mentioned three locations over 2–3 years. By statistical analysis, we determined the IHT for the two hybrids.

## Materials and Methods

### Plant materials and experimental design

Maize hybrid ZD958 was developed in 1996 by the Henan Academy of Agricultural Science, Henan Province; XY335 was developed in 2000 by the Pioneer Technology Co., Tieling, Jilin Province, China. Both varieties had middle to late maturity with a growth period of approximately 130 days.

Field experiments were conducted over 2–3 consecutive years (2013–2015) in China at Lanzhou (LZ), Gansu province (36°03′N, 103°49′E), Urumqi (Um), Xinjiang Uygur autonomous region (44°03′N, 87°19′E) and Shenyang (SY), Liaoning province (41°48′N, 123°23′E; Table 1). The three plots above are the typical seed-producing areas that provide more than half of the hybrid maize seed product in China.

**Table 1:** Ecological conditions and plant growth statuses in Lanzhou (LZ), Urumqi (UM) and Shenyang (SY) over 2–3 years (2013, 2014 and 2015).

location	Year	Position	Sunshine duration in August (h)	Annual precipitation (mm)	Planting data	pollination date	
					XY335/ZD958	XY335	ZD938
LZ	2013	103°49′E, 36°03′N	12:56–13:58	255.5	April 20	July 25	July 22
	2014			355.6	April 24	July 25	July 22
UM	2013	87°19′E, 44°03′N	13:12–14:35	300.9	May 1	July 30	July 27
	2014			297.0	May 4	July 26	July 23
	2015			408.9	May 6	July 31	July 28
SY	2013	123°23′E, 41°48′N	13:08–14:27	788.1	April 25	July 24	July 24
	2014			362.9	April 28	July 29	July 26
	2015			573.2	April 28	July 30	July 27

### Measurements

#### Seed production

Each hybrid was planted in an isolation plot at one environment by using a randomized complete block design (RCBD) with three blocks. Field plots were over-seeded by hand broadcast but had a final stand of 60,000 female seedlings and 15,000 male seedlings ha^−1^, which is the optimal population density for both XY335 and ZD938 seed production, by hand thinning at the 3-leaves-stage. Plots were planted with 4 female rows separated by 1 male row. The row spacing was 65 cm and the plant distance within one row was 20 cm. All the females were hand-detasseled before tassel emergence to exclude the contamination of self-pollination. After open pollination, the male plants were cut and removed from the field to expand the space for the female plants. The harvesting was conducted from 33 DAP (days after pollination) until 63 DAP in 3-day intervals. For each harvest, ears were randomly harvested from the two center rows of the four female row plots in each field block.

Plots were fertilized with 85 kg ha^−1^ P_2_O_5_, 90 kg ha^−1^ K_2_O and 100 kg ha^−1^ nitrogen (217 kg ha^−1^ urea) before sowing. An additional 60 kg ha^−1^ nitrogen was used to fertilize the plants at the shooting stage (6 leaves) and the silking stage, along with timely irrigation. Turf machinery and weeding were applied before sowing to ensure seedling establishment. Annual precipitation in the above three locations was generally less than 600 mm (http://www.stats.gov.cn), which was not sufficient for maize growth. Thus, all the fields were sprinkler-irrigated for sufficient water supply and to exclude the possibility of water effects on seed quality.

The information on planting dates and pollination data as well as growing conditions for each seed production environment are shown in Table 1. The ears that had uniform appearance were selected and tagged for later sampling. The pollination time was defined as the day that 50% of the tagged plants within one plot were anthering.

#### Treatment evaluation

SMC was determined according to the international seed testing association (ISTA) rules^4^. Briefly, 150 seeds were randomly counted from the middle parts of fresh harvested ears (5 ears and 30 seeds per ear were collected). The seeds were weighed and prebaked (first drying) at 105 °C for 5 hr in an oven. The water loss was calculated and denoted as percentage S1. After grounding into a powder, a subsample (5 g/sample) was dried again (second drying) at 130 °C for 1 hr. The water loss from the second drying was expressed as S2. The SWC was calculated according to the formula below:

$${\rm{SWC}}={\rm{S1}}+{\rm{S2}}-{\rm{S1}}\times {\rm{S2}}/{\rm{100}}$$

The HSW was calculated using data from the SMC measurement and standardized to 14% moisture.

For consistency, the 10 fresh-harvested ears were naturally dried and hand-shelled, followed by seed vigor testing according to the ISTA rules^4^. SG was conducted with paper towels by placing 50 seeds on top of a paper towel and rolling it into a column and then incubating at 25 °C for 7 days. The germinated seeds were counted, and SG was expressed as the percentage of germinated seeds. AAG was conducted by placing 50 seeds in sealed plastic boxes, which were then placed in a thermostatic moisture regulator (Thermoline Scientific, NSW, Australia. Plant growth cabinet 1100 L) under a regime of 45 ± 1 °C and 85–95% relative humidity. The germination percentage of the aged seeds was immediately determined by the same method as the above SG test. CTG was performed by placing 50 seeds in a prechilled (10 °C) cold test chamber. The paper towels prepared by the SG method were incubated for 7 days at 10 °C and then 4 days at 25 °C. The SG, AAG and CTG tests were replicated 4 times for each harvest.

The AT10_ph_ for seed development is the sum of the mean daily temperatures during the growing period from the pollination day to the harvest day for each hybrid within one environment in which the mean daily temperature is above 10 °C^21,22^. The calculation equation is as follows:

$$\mathrm{AT}10=\sum _{n=1}^{n}[\frac{({\rm{T}}\max +T{\min })}{2}]$$

where Tmax and Tmin are the maximum temperature and minimum temperature in each day, respectively; n is the days after pollination. AT10 is set equal to 0 if it is less than 10 °C.

### Statistical Analyses

The data with replicates across genotype, harvest stage, location and year were pooled for analysis of variance (ANOVA) with a four-factor variance analysis program by using the GLM program in the SAS software (SAS Institute, 1993; Cary, NC, USA). Differences were compared using the least significant difference test (LSD) at the 0.05 level of probability. Principal coordinate analysis (PCA) was also calculated in the SAS software by using the PRINCOMP program.

The trait response curves to harvest stages were generated using the NLIN procedure in SAS. Three response models were evaluated: linear, quadratic, and linear-with-plateau^27^. The best-fit data are reported here, and the calculated optimal harvest time for each genotype are provided.

## Results

### Relationship of seed vigor, physiological and ecological traits

**Figure 1:**
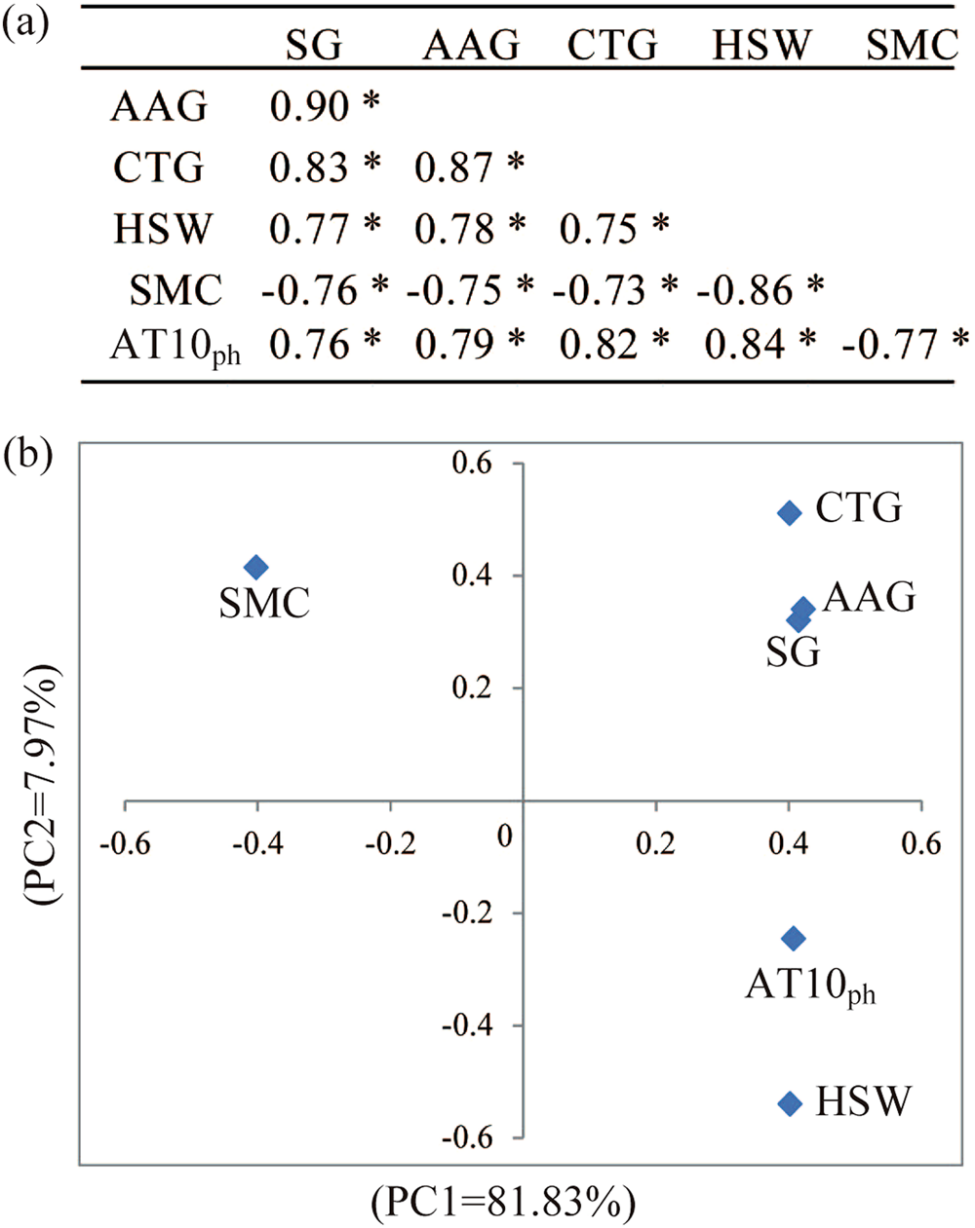
Relationships of seed-related traits. (**a**) Correlation between each pair of the investigated traits. (**b**) Principle component analysis (PCA) of the investigated traits. Seeds were collected at 11 different growth stages that were generated from 8 environments to evaluate three vigor traits (standard germination, SG; accelerated aging germination, AAG and cold test germination, CTG), two physiological traits (hundred-seed weight, HSW and seed moisture content, SMC), and an ecological trait (≥10 °C accumulated temperature from pollination to harvest, AT10_ph_). *Indicates a significant correlation at p < 0.001.

A total of six seed-related traits, including three vigor traits (SG, AAG and CTG), two physiological traits (HSW and SMC) and one ecological trait (AT10_ph_), for hybrids ZD958 and XY335 was collected at 11 harvest stages under 8 environments (Table 1). As indicated by Pearson correlation coefficients (Fig. 1), the three seed vigor traits showed significant positive correlations with HSW and AT10_ph_, while they were negatively correlated with SMC. SG showed a similar correlation value with HSW, SMC and AT10_ph_ (R = 0.76–0.77), while AAG and CTG showed higher correlations with AT10_ph_ (R = 0.79–0.82) than with HSW and SMC (R = 0.73–0.78). Principle component analysis (PCA) showed that the three seed vigor traits were all closely related and grouped together (Fig. 1). AT10_ph_ had a closer relationship to the seed vigor group than what the SMC and HSW demonstrated. Above all, both the Pearson correlation coefficient and PCA suggested closer relationships of seed vigor traits to AT10_ph_ in maize.

### ANOVA results for seed vigor, physiological and ecological traits

The ANOVA results showed that SG, AAG, CTG, HSW and SMC were significantly affected by variety, location, year and harvest stage, with an exception of HSW by variety (Table 2). These five traits were also significantly affected by interactions of year × harvest stage and location × harvest stage, indicating that the effects of harvest stage on these traits varied among different locations and years. Meanwhile, AT10_ph_ was the only trait significantly affected by location and harvest stage (Table 2).

**Table 2:** Performance and ANOVA of six seed-related traits collected in seeds from Lanzhou (LZ), Urumqi (UM) and Shenyang (SY) over 2–3 years (2013, 2014, and 2015). Seeds were harvested at 11 stages from 33 days after pollination (DAP) to 63 DAP to evaluate traits of standard germination (SG), accelerated aging germination (AAG), cold test germination (CTG), hundred seed weight (HSW), seed moisture content (SMC) and ≥10 °C accumulated temperature from pollination to harvest (AT10_ph_).

Source of variance	SG (%)	AAG (%)	CTG (%)	HSW (g)	SMC (%)	AT10ph (°C)
**Variety**						
XY335	87.52 ± 12.82 a^1^	81.98 ± 15.23 a	76.77 ± 19.21 a	28.07 ± 5.58 a	37.63 ± 6.80 b	1058.07 ± 176.04 a
ZD958	85.19 ± 14.68 b	81.14 ± 14.54 b	72.44 ± 23.06 b	27.98 ± 6.42 a	41.43 ± 9.09 a	1075.15 ± 180.73 a
**Location**						
LZ	80.85 ± 19.56 c	78.80 ± 18.57 c	71.64 ± 21.84 b	24.56 ± 5.92 c	44.65 ± 8.28 a	1055.73 ± 183.96 b
UM	85.43 ± 11.75 b	80.18 ± 14.88 b	69.28 ± 23.45 c	29.86 ± 4.99 a	37.83 ± 8.04 b	1037.58 ± 166.79 b
SY	90.94 ± 8.77 a	84.78 ± 11.15 a	81.78 ± 16.29 a	28.48 ± 6.05 b	37.80 ± 6.93 b	1102.90 ± 181.44 a
**Year**						
2013	87.82 ± 11.24 a	84.14 ± 13.05 a	78.96 ± 20.16 a	27.30 ± 5.73 c	38.57 ± 8.75 b	1098.16 ± 179.69 a
2014	84.93 ± 16.89 c	80.84 ± 15.34 b	72.52 ± 20.32 b	28.86 ± 6.16 a	41.02 ± 8.32 a	1062.64 ± 176.18 ab
2015	86.35 ± 11.95 b	79.01 ± 16.10 c	71.49 ± 23.36 b	27.86 ± 6.06 b	38.71 ± 7.07 b	1028.42 ± 174.31 b
**Harvest stage (Days after pollination)**						
33	61.24 ± 16.66 g	54.21 ± 15.74 h	34.50 ± 13.10 i	18.14 ± 3.27 j	51.73 ± 5.97 a	781.43 ± 37.25 j
36	70.89 ± 14.85 f	64.75 ± 13.14 g	48.54 ± 16.88 h	20.57 ± 3.03 i	49.06 ± 4.85 b	844.72 ± 38.67 ij
39	78.54 ± 10.87 e	73.60 ± 10.27 f	59.42 ± 17.00 g	23.09 ± 3.46 h	45.41 ± 5.00 c	904.81 ± 38.15 hi
42	84.43 ± 7.19 d	79.85 ± 8.99 e	66.80 ± 15.93 f	26.33 ± 3.87 g	43.31 ± 4.37 d	963.34 ± 38.97 gh
45	89.60 ± 4.95 c	81.77 ± 8.97 e	78.17 ± 11.21 e	28.07 ± 3.25 f	41.42 ± 4.10 e	1021.34 ± 38.01 fg
48	92.30 ± 4.78 b	87.44 ± 7.02 d	83.15 ± 8.42 d	29.47 ± 3.02 e	38.50 ± 4.10 f	1077.31 ± 39.92 ef
51	93.34 ± 4.62 b	90.35 ± 5.18 bc	87.33 ± 6.42 c	30.74 ± 3.01 d	37.03 ± 3.86 g	1130.34 ± 49.91 de
54	94.85 ± 4.07 a	91.65 ± 5.20 ab	90.33 ± 4.88 ab	32.60 ± 2.75 c	34.94 ± 3.33 h	1180.28 ± 49.15 cd
57	94.79 ± 3.57 ab	92.35 ± 4.13 a	90.93 ± 6.27 a	33.12 ± 2.63 b	32.96 ± 4.44 i	1230.00 ± 56.61 bc
60	95.46 ± 3.07 a	91.68 ± 4.61 ab	89.38 ± 6.06 abc	33.29 ± 2.35 b	31.36 ± 4.49 j	1277.91 ± 64.88 ab
63	94.46 ± 3.48 ab	89.50 ± 5.34 c	88.58 ± 5.82 bc	33.70 ± 2.23 a	31.00 ± 6.05 k	1321.25 ± 71.16 a
**Source of variation**						
Variety (V)	743.02 (1)***^2, 3^	224.52 (1)***	3287.31 (1)***	1.43 (1)	1944.08 (1)***	12275.08 (1)
Location (L)	4079.83 (2)***	2116.14 (2)***	9346.66 (2)***	1141.28 (2)***	2080.87 (2)***	83270.22 (2)*
Year (Y)	435.59 (2)***	1102.84 (2)***	2546.39 (2)***	251.24 (2)***	202.32 (2)***	46975.88 (2)
Harvest stage (H)	6555.68 (10)***	7859.73 (10)***	16952.65 (10)***	1364.82 (10)***	2419.30 (10)***	488276.72 (10)**
V*L	639.28 (2)***	903.09 (2)***	1077.21 (2)***	22.61 (2)***	38.33 (2)***	1154.84 (2)
V*Y	226.90(2)***	260.60 (2)***	111.13 (2)**	34.53 (2)***	19.28 (2)***	364.99 (2)
V*H	28.51 (10)*	19.58 (10)	160.59 (10)**	9.27 (10)***	52.98 (10)***	196.30 (10)
V*L*Y	54.62 (6)*	93.15 (6)**	220.38 (6)***	41.91 (10)***	50.21 (6)***	495.60 (3)
L*H	433.83 (20)***	257.47 (20)***	452.23 (20)***	8.86 (20)***	6.26 (20)***	945.62 (20)
Y*H	88.95 (20)***	83.45 (20)***	63.90 (20)***	3.47 (20)***	6.82 (20)***	441.75 (20)
L*Y*H	113.71 (29)***	141.79 (29)***	88.21 (29)***	10.79 (29)***	14.21 (29)***	420.72 (29)
V*L*H	73.37 (20)***	31.97 (20)	62.38 (20)***	4.85 (20)***	16.68 (20)***	32.31 (20)
V*Y*H	29.75 (20)**	64.28 (20)***	60.71 (20)***	5.42 (20)***	9.69 (20)***	31.38 (20)
V*L*Y*H	22.12 (29)*	61.73 (29)***	112.34 (29)***	5.94 (29)***	8.67 (29)***	25.40 (29)
Rep	8.00 (2)	18.84 (2)	6.60 (2)	0.29 (2)	0.58 (2)	—
Error	13.91 (350)	23.37 (352)	28.44 (347)	0.97 (342)	0.59 (350)	2493.81

^1^The numbers followed by different letters indicate significant differences (P < 0.05) within variety, harvest stage, location or year.

^2^The number in parentheses indicates the degrees of freedom (df) for the variance.

^3^*, ** and *** following the number represent significance at p ≤ 0.05, p ≤ 0.01 and p ≤ 0.001, respectively.

Among the factors that affected the investigated seed traits, harvest stage was the most important factor that impacted all traits. Following harvest stage, the factor location had larger effects than year or variety on all investigated traits (Table 2). These results suggested that all six seed-related traits were the most sensitive to different harvest stages, moderately sensitive to different locations, and the least sensitive to different years.

### Performance of seed vigor, physiological and ecological traits in different environments

All three seed vigor traits (SG, AAG and CTG) in the SY location had the highest values, which were 12.48%, 7.59%, and 14.15% and 6.45%, 5.74%, and 18.04% higher than that in LZ and UM, respectively. The higher seed vigor in SY was probably due to the higher AT10_ph_ obtained at this location (Table 2). The seed physiology trait HSW in LZ was 21.58% and 15.96% lower than that in UM and SY, respectively, and SMC was 18.03% and 18.12% higher than that in UM and SY, respectively. The lower HSW and higher SMC in LZ might result from the duration of shorter sunshine in the southern area in comparison to the other two northern locations (Table 1).

Among the three years, SG, AAG and CTG in 2013 were 3.40%, 4.08% and 8.88%, and 1.69%, 6.49% and 10.45% higher than that in 2014 and in 2015, respectively, which also probably due to the higher AT10_ph_ obtained in 2013 (Table 2).

### Response of seed-related traits to harvest stage

When the two hybrids were combined, the coefficient of variance (CV) for the three seed vigor traits were higher at early harvest stages than that at later stages. The average CV for the first 5 harvests were 15.21%, 17.10% and 27.89% for SG, AAG and CTG, while that for the later 5 harvests were only 4.02%, 5.83% and 7.15%, respectively (Table 2). A higher CV at the early harvest stages indicated that the immature seeds were more sensitive to environmental changes than the mature seeds. Meanwhile, the CV for HSW, SMC and AT10_ph_ were relatively stable during all harvest stages.

From the ANOVA, SG increased from the first harvest and was maintained at a plateau after 54 DAP (Table 2). AAG and CTG increased first, reached their peaks at 54 DAP, and then slightly decreased at 63 DAP. These results indicated that the highest seed vigor could be obtained at 54 DAP to 60 DAP. Meanwhile, HSW and AT10_ph_ increased, and SMC decreased all through the harvests across 8 environments (Table 2).

**Figure 2:**
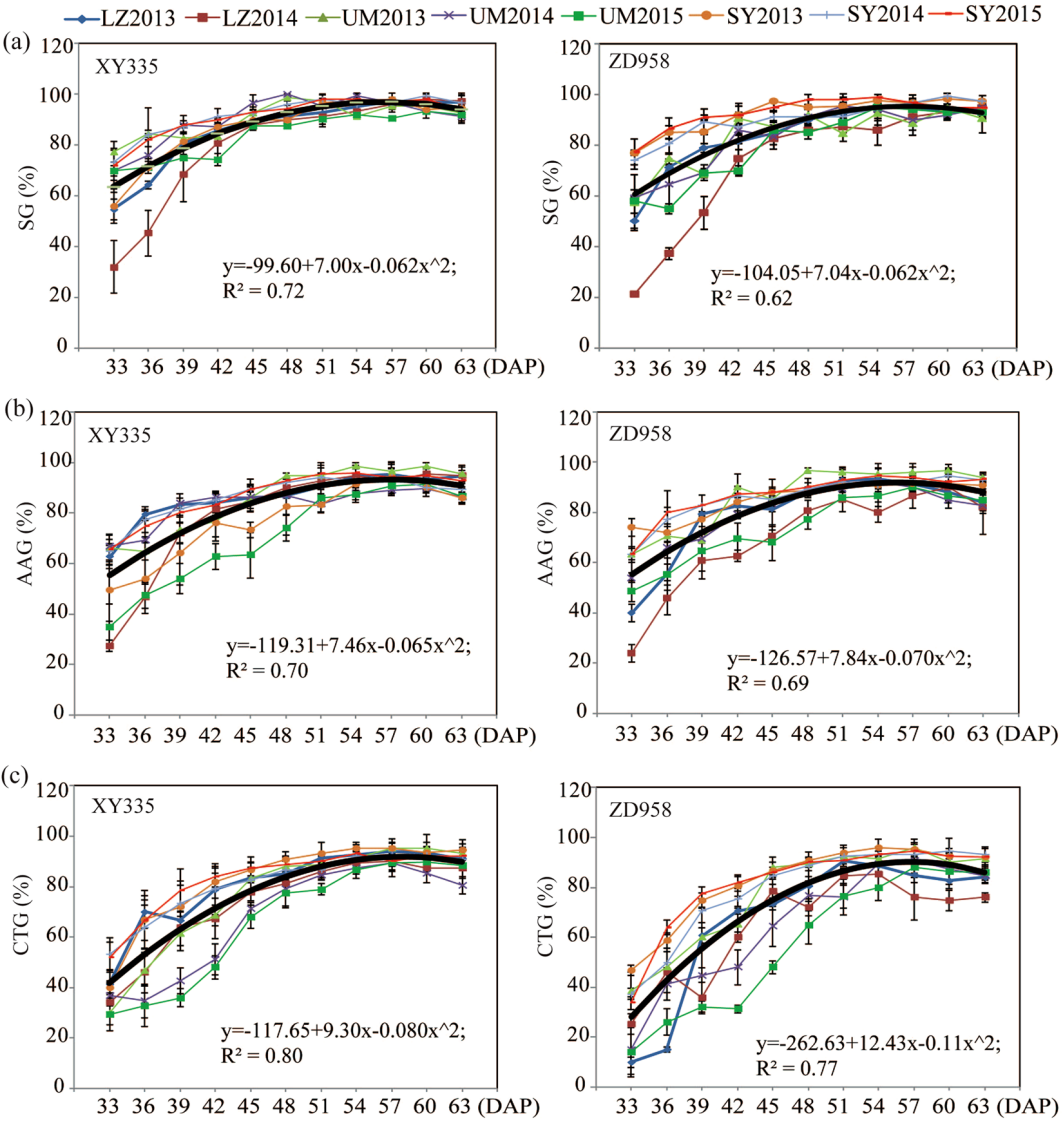
Performance of (**a**) standard germination (SG), (**b**) accelerated aging germination (AAG) and (**c**) cold test germination (CTG) in response to harvest stage in XY335 and ZD958 seeds grown in 3 locations (LZ, UM and SY) over 2–3 years (2013, 2014 and 2015). The relationship between harvest stage and the investigated traits was simulated using the “quadratic”, “linear” and “linear-plateau” models in the SAS program. The results from the best-fit model are presented by black lines and equations.

**Figure 3:**
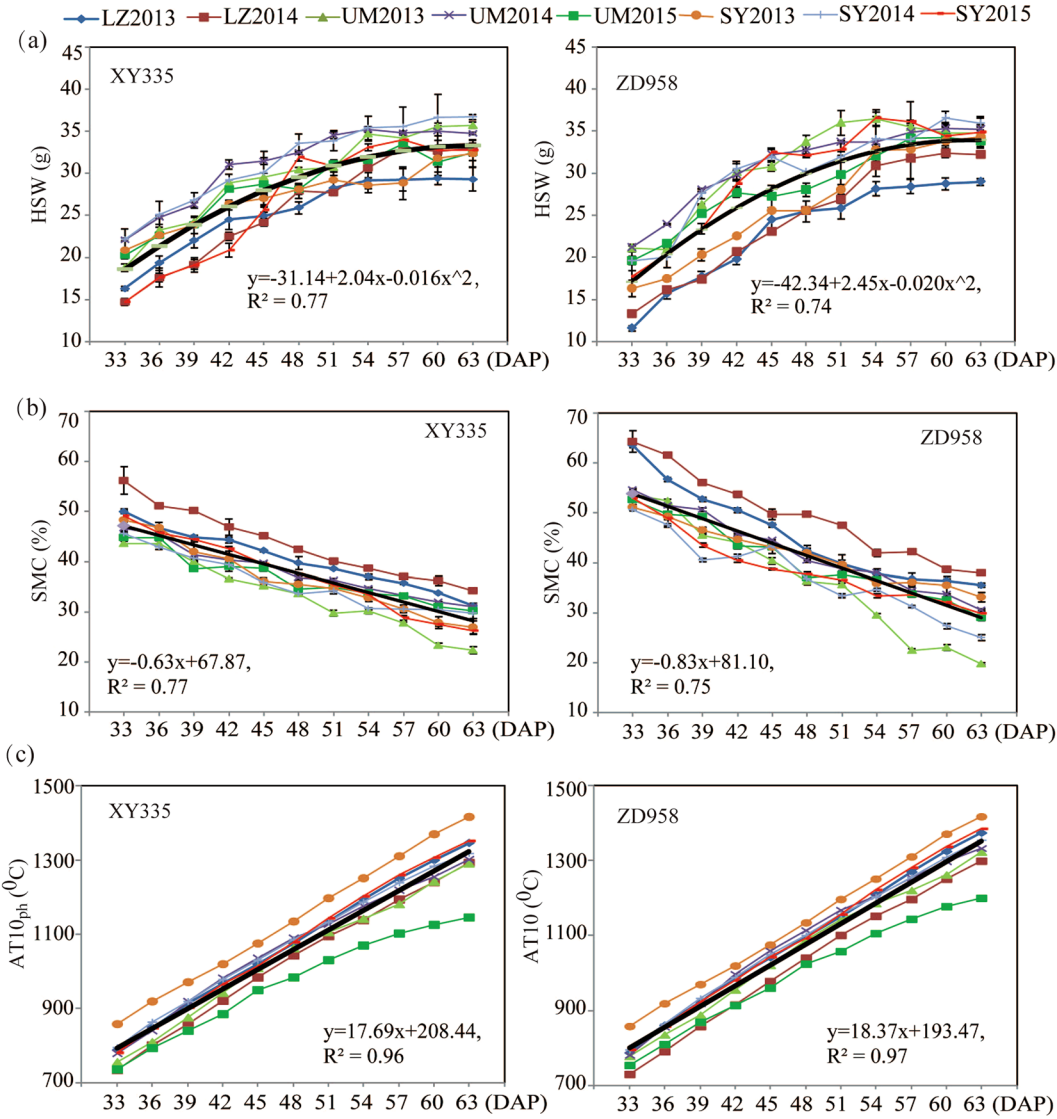
Performance of (**a**) hundred-seed weight (HSW), (**b**) seed moisture content (SMC) and (**c**) ≥10 °C accumulated temperature from pollination to harvest (AT10_ph_) in response to harvest stage in XY335 and ZD958 grown in 3 locations (LZ, UM and SY) over 2–3 years (2013, 2014 and 2015). The relationship between harvest stage and the investigated traits was simulated using the “quadratic”, “linear” and “linear-plateau” models in the SAS program. The results from the best-fit model are presented by black lines and equations.

To find the IHT for XY335 and ZD958, we further used statistical models to fit the relationships between harvest stage and the seed vigor traits in each hybrid. The relationships in both hybrids were similar and were fitted to a quadratic model with satisfactory R^2^ ranging from 0.62 to 0.80 (Fig. 2). HSW was also fitted to a quadratic model (R^2^ = 0.74–0.77; Fig. 3). However, the variables of SMC appeared to have a decreasing linear tendency, and the AT10_ph_ showed an increasing linear tendency (Fig. 3).

According to the quadratic behaviors, the maximum value for each equation could be found. The periods required to achieve maximum SG, AAG, and CTG were 56.17 DAP, 57.01 DAP and 57.97 DAP for XY335, respectively, and 56.62 DAP, 55.73 DAP and 56.80 DAP for ZD958, respectively (Table 3). To obtain high-quality seed, the last date to achieve maximum SG, AAG or CTG for each hybrid was set as its IHT. Therefore, the IHTs for XY335 and ZD958 were 57.97 DAP and 56.80 DAP, respectively (Table 3). The corresponding SMC and AT10_ph_ at the IHT were 33.15% and 1234 °C for XY335, and 34.98% and 1225 °C for ZD958 (Table 4). Meanwhile, the period required for reaching PM was 63.28 DAP and 62.24 DAP for XY335 and ZD958, respectively (Table 4 & 5). Thus, the interval between IHT and PM was 5.31 and 5.44 days in XY335 and ZD958, respectively (Table 4), suggesting that both seeds could be harvested to maintain high vigor at approximately 5 days before they reached PM.

**Table 3:** Maximum values of hundred-seed weight (HSW), standard germination (SG), accelerated aging germination (AAG) and cold test germination (CTG), and the corresponding days from pollination to the predicted date for achieving maximum levels in XY335 and ZD958 across 8 environments.

Variety	HSW (g)		SG (%)		AAG (%)		CTG (%)		IHT^2^
	maximum level	DAP to maximum level^1^	maximum level	DAP to maximum level	maximum level	DAP to maximum level	maximum level	DAP to maximum level	
XY335	33.33	63.28	97.00	56.17	93.25	57.01	91.89	57.97	57.97
ZD958	33.97	62.24	95.33	56.62	91.77	55.73	90.26	56.80	56.80

^1^The corresponding DAP (days after pollination) indicate days from pollination to the predicted dates at which the maximum level of each trait will be reached.

^2^IHT, the ideal harvest time, the last date to achieve maximum SG, AAG and CTG.

**Table 4:** Seed moisture content (SMC) and >10 °C accumulated temperature from pollination to harvest (AT10_ph_) at the ideal harvest time (IHT) and physiological maturity (PM) point in XY335 and ZD958 across 8 environments.

Variety	IHT^1^	PM point^2^	Interval between IHT and PM	SMC (%)		AT10_ph_ (°C)	
				IHT	PM	IHT	PM
XY335	57.97	63.28	5.31	33.15	29.99	1234	1330
ZD958	56.80	62.24	5.44	34.96	30.46	1226	1326

^1^IHT, the ideal harvest time, the last date to achieve maximum SG, AAG and CTG.

^2^PM point, the DAP to achieve the maximum HSW level.

## Discussion

The hybrid maize seed industries are increasingly conscious of the necessity of increasing the quality of the seeds they produce. SG, CTG and AAG are the three most important indexes to predict seed quality^4^. Previous investigations found that maize seed quality could be greatly influenced by the percentage of seed maturity, which is greatly affected by different harvest times^10,20^. However, most investigations only used SG as a seed vigor index and seldom comparisons of seed maturity to CTG and AAG vigor could be addressed, except that TeKrony and Hunter conducted the CTG test and Junior *et al*. conducted the CTG and AAG tests to investigate the relationship between seed vigor and seed maturity^10,20^. In this work, we conducted SG, AAG and CTG tests for maize hybrid seeds harvested from 11 growth stages across 8 environments. More harvest stages and growth environments together with the three seed vigor indicators provided the possibility to conduct a comprehensive analysis of the IHT for the hybrid maize XY335 and ZD958.

First, we found that the maximum SG, CTG and AAG were obtained at the same maturity stage in the hybrids ZD958 and XY335 (Fig. 2 and Table 3). A similar stage for achieving maximum SG and CTG has also been observed in previous work, while it was observed that obtaining the maximum AAG took several days longer^10^. The different dates to achieve maximum SG and AAG might result from the different genotypes used in these studies. After designating the last date to achieve maximum SG, AAG and CTG as IHT; the IHT for XY335 and ZD958 were 57.97 DAP and 56.80 DAP, respectively (Table 3).

Second, we observed that the IHT occurred approximately 5 days earlier than the PM point in both XY335 and ZD958 (Table 4). This result was supported by several previous studies^10,15,20^. However, one study had another hypothesis that the IHT occurred as close as possible to the PM point^28^. Junior *et al*. (2014) imputed this difference to the non-uniformity seed maturity process in the field, where maximum seed vigor was obtained when most of the seeds in the population reached maturity, but a few seeds still accumulated dry matter^10^. However, for a single seed or a uniform seed lot, the stage to achieve the IHT was the same as that to reach PM. In this work, seeds might have been uniform when they were randomly selected from a whole ear. Further experiments with self-pollination and more precise harvesting (e.g., harvesting seeds only from the middle part of an ear) might be required to provide a conclusive result. On the other hand, we observed that seed nutritional parameters such as protein content, starch content, oil content and soluble sugar content reached a plateau after the IHT point (data not shown), suggesting that seed chemical quality was similar between IHT and PM points. The difference in seed vigor between these two points might be a result of other unknown factors, such as seed deterioration.

Third, across 8 environments, we found that XY335 and ZD958 seeds were harvested at SMC levels of 33.15–34.98% (Table 4). These results were similar to previous studies. Kinittle and Burris reported that single cross seeds reached a maximum seed vigor (as measured by shoot and root weight) at an SMC level ranging from 33.1% to 37.3%^17^. Rush and Neal concluded that double cross seeds achieved maximum CTG at approximately 35% SMC^29^. Thus, these works confirmed that seeds reached the IHT at a stable SMC level of approximately 35%.

Fourth, we found that AT10_ph_ might be a good indicator for seed maturity and seed harvest. Seed dry weight was previously suggested as a reliable indicator for seed vigor^19,20^. However, we found that the maximum seed vigor occurred a few days before the seeds reached PM, suggesting that seeds can be harvested before the PM point. In addition, HSW increased slowly when seed growth was close to the PM point, which might result in a large variation of HSW in determining the PM point. Thus, an indicator other than HSW will be desired. AT10_ph_ was less affected by variety, year and location in comparison with HSW and SMC (Table 2). In addition, AT10_ph_ showed a closer relationship to the seed vigor indexes than SMC and HSW (Fig. 1), suggesting that AT10 was a reliable parameter for indicating seed vigor. Since AT10 is routinely used to determine crop planting schedules, crop varieties and crop patterns^22–25^, AT10_ph_ used as an indicator for seed production might be effective. For both hybrids XY335 and ZD958, the seeds reached the IHT when AT10_ph_ reached 1226–1234 °C (Table 4).

## Conclusions

Across 8 environments, we found that seeds of XY335 and ZD95 reached their IHT at 57.97 DAP and 56.80 DAP, respectively, with a corresponding SMC of 33.15% and 34.98%. The date to reach IHT is approximately 5 days earlier than that for reaching the PM point, suggesting that both hybrid seeds could be harvested before the PM point. AT10_ph_ was suggested as a better indicator for determining the IHT. For both hybrids, the ideal harvest AT10_ph_ was 1226–1234 °C.
